# EFFECT OF AMYLOID PET RESULTS DISCLOSURE ON HEALTH-RELATED BEHAVIORS IN PERSONS WITH MILD COGNITIVE IMPAIRMENT

**DOI:** 10.1093/geroni/igad104.1566

**Published:** 2023-12-21

**Authors:** Yan Wang, Dianxu Ren, Scott Roberts, Jennifer Lingler

**Affiliations:** School of Nursing University of Pittsburgh, Pittsburgh, Pennsylvania, United States; University of Pittsburgh, Pittsburgh, Pennsylvania, United States; University of Michigan, Ann Arbor, Michigan, United States; University of Pittsburgh, Pittsburgh, Pennsylvania, United States

## Abstract

Mounting evidence supports the clinical utility of amyloid PET. However, whether patients use knowledge of their amyloid status to alter health behaviors remains unclear. We describe health-related actions taken by Mild Cognitive Impairment patients following amyloid PET disclosure (n=34) vs. a comparison group (n=37). Patients were 92% non-Hispanic white, 59% male, 73±8.61 years old with 16.25±2.49 years of education. Over 12 months of follow-up, all participants reported at least one behavior change from baseline on a 14-item health behavior questionnaire. Amyloid positive patients reported the most behavior changes (mean=4.67±1.83) while amyloid negative patients reported the fewest (mean=3.72±1.58). Across groups, no significant differences were observed in: lifestyle, vitamin/supplement use, stress reduction activities, cognitive stimulation, or advance directive completion. Amyloid negative participants were significantly less likely than controls to consider long-term care insurance (63.6% vs. 89.2%; P=.025), and to endorse changes classified as “other” (36.4% vs. 64.9%; P=0.037). After adjusting for education level, gender, and MMSE, logistic regression showed that amyloid negative patients were 74% less likely than controls to report “other” changes (OR=0.26, 95% CI [0.08, 0.85], P=0.025), and 78% less likely to consider long-term care insurance (OR=0.22, 95% CI [0.06, 0.86], P=0.03). Qualitative analysis of supplemental interviews with scan group participants revealed “other” activities to include changes in employment, driving, and residential status, and engagement in other non-medical activities (pursuing bucket lists and mending strained relationships). Health-related behavior changes following amyloid PET disclosure may differ by scan result and encompass actions to enhance not only cognition but quality of life.

